# A comprehensive systematic review of randomized controlled trials on anesthetic agents in children’s upper gastrointestinal endoscopy: highlighting safety concerns and efficacy

**DOI:** 10.1007/s00210-025-04557-2

**Published:** 2025-09-04

**Authors:** Amr Elrosasy, Mahmoud Diaa Hindawi, Mohamed Abo Zeid, Abdelaziz A. Awad, Ahmed W. Abbas, Mohammad Al Diab Al Azzawi, Eslam Afifi, Ahmed Amgad, Mohamed Yasser, Khalid Sarhan, Sara Chikh Aissa

**Affiliations:** 1 Faculty of Medicine, Cairo University, Cairo, Egypt; 2 Faculty of Medicine, Al-Azhar University, Cairo, Egypt; 3 Faculty of Medicine, Tanta University, Tanta, Egypt; 4 Medical Research Group of Egypt, Negida Academy, Arlington, MA USA; 5 Faculty of Medicine, Mansoura University, Mansoura, Egypt; 6 Faculty of Medicine, National Ribat University, Khartoum, Sudan; 7 Faculty of Medicine, Benha University, Benha, Egypt; 8 Faculty of Medicine, Helwan University, Cairo, Egypt; 9 Mohamed Lamine, Debaghine University Hospital, Algiers, Algeria

**Keywords:** Pediatric gastrointestinal disorders, Upper gastrointestinal endoscopy (GIE), Sedation, Remimazolam, Propofol, S-Ketamine

## Abstract

Upper gastrointestinal endoscopy (GIE) is essential for diagnosing and treating gastrointestinal disorders in children aged 6–12 years, yet it often requires sedation due to the significant discomfort and pain involved. We conducted a PRISMA 2020–compliant systematic review of randomized controlled trials (RCTs) from PubMed, Web of Science, Scopus, and Ovid (inception to March 30, 2024). Inclusion criteria are as follows: RCTs comparing sedative regimens (e.g., propofol, ketamine, remimazolam, dexmedetomidine) in children undergoing upper GIE. Exclusion criteria are as follows: non-RCTs, studies outside the age range, or non-English publications. Risk of bias was assessed using Cochrane ROB-2. Data were extracted for recovery time, hemodynamic parameters, and adverse events (hypoxia, bradycardia, dizziness). A systematic synthesis of outcomes was performed, with results presented descriptively and quantitatively (e.g., event rates, mean differences) to compare regimens. Nineteen RCTs were included with a total of 1955 patients. Propofol, either alone or in combination, was frequently used. The propofol-ketamine combination showed better hemodynamic stability (92.2 ± 16.8 bpm) compared to propofol-fentanyl (76.8 ± 13.8 bpm). S-ketamine demonstrated dose-dependent effects—0.3 mg/kg provided the shortest recovery time (33.5 min) with moderate dizziness (40.0%)—while 0.5 mg/kg offered optimal heart rate maintenance (93.81 bpm) but longer recovery (35.67 min) and increased dizziness (43.3%). The 0.7 mg/kg dose showed faster recovery than 0.5 mg/kg (33.5 vs 35.67 min), but the highest dizziness rates (73.3%). Post-procedural complications were minimal except for dose-dependent neurological effects with S-ketamine (visual disturbances peaking at 27.6% with 0.3 mg/kg). Remimazolam showed the fastest recovery overall. Adverse events varied by regimen: propofol-ketamine had higher hypoxia (6.8%) and dizziness (34.1%), while propofol-fentanyl showed more bradycardia (24.4%). Overall, remimazolam and dexmedetomidine regimens were linked to fewer complications, though they required careful monitoring for hypotension. However, heterogeneity in outcomes (e.g., recovery times, adverse events) underscores the need for individualized regimen selection. Limitations include variability in study designs and insufficient data on minimal effective doses. Further RCTs should standardize outcome measures and optimize dosing for children undergoing endoscopy.

**Supplementary Information:**

The online version contains supplementary material available at 10.1007/s00210-025-04557-2.

## Introduction

Gastrointestinal endoscopy (GIE) is one of the main procedures that is widely used as a diagnostic or therapeutic intervention in the treatment of various gastrointestinal tract (GIT) disorders in children (ages 6–12) within current clinical practice (Oliva S. et al., 2017). Although GIE is a highly efficient intervention, it is still fundamentally a painful procedure with many complications, such as abdominal pain and bloating in lower GIE procedures such as colonoscopy, choking, and retching in upper GIE procedures, such as esophagogastroduodenoscopy (EGD) (Attard TM et al., 2018; Ghanouni A. et al., 2016). The application of GIE in children is more difficult than that in adults because children tend to be less comfortable and cooperative, which increases agitation during the procedure, leading to more complications (Isoldi S. et al., 2021).

To overcome the side effects of GIE, sedation has been a major part of most of the procedures in developed countries (Lichtenstein DR et al., 2008). The addition of sedation has increased the success rate of the procedures and reduced the rate of related side effects, making it easier for both the patient and the doctor to conduct the procedure (Oliva S. et al., 2017; Lin OS, 2017).

A universally accepted sedation formula for GIE does not exist, yet various regimens are employed, reflecting a diversity in approaches (Rana MV et al., 2016). The most commonly used regimen is a combination of a short-acting hypnotic agent and analgesic(Rana MV et al., 2016; Childers RE et al., 2015). The hypnotic agent is usually either propofol or a benzodiazepine. The use of propofol tends to be more common due to its efficacy and rapid onset of action (Early DS et al., 2018); however, despite being relatively safe, it is still associated with cardiovascular (CVS) and respiratory depression, leading to major symptoms such as hypotension and apnea (Coté GA et al., 2010; Goudra B et al., 2020). Remimazolam is a novel benzodiazepine drug that could be a replacement for propofol and is reported to have a similar efficacy and lower major adverse effects compared to propofol (Barbosa EC et al., 2024).

While the hypnotic agent is responsible for sedation, the analgesic drug’s role is to reduce pain during the procedure as well as increase the potency of the sedating drug (Lichtenstein DR et al., 2008; Early DS et al., 2018). Opioids are still the most used analgesics in GIE, but despite their proven efficacy, their addition to propofol further exacerbates the incidence of CVS and respiratory depression (Shetabi H et al., 2018). Many alternatives to opioids have been examined in the literature, such as ketamine and dexmedetomidine, each possessing its advantages and drawbacks (Hu Z et al., 2022; Amer et al., 2020).

The importance and widespread use of upper GIE procedures in children urge us to explore the various regimens and the different drugs used in the sedation process to further explain the differences between each drug and demonstrate the efficacy and safety of each regimen. Therefore, we aimed to investigate this topic by conducting this systematic review.

## Methods

The methods and outcomes of our study adhered closely to the recommendations for systematic reviews and meta-analyses, which included applying PRISMA 2020 (Page MJ et al., 2021) and the Cochrane Handbook (2019), Prospero registration (CRD42024583882).

### Literature search

In accordance with the PRISMA guidelines, we conducted an exhaustive search across several databases, including PubMed, Web of Science (WOS), Scopus, and Ovid databases. This investigation included key terms, including ketamine, propofol, and upper gastrointestinal endoscopy. The period was covered from the first records in the databases to March 30, 2024. ESM. 1 contains a comprehensive strategy. The retrieved references were then imported into the Rayyan software (Ouzzani M et al., 2016) for the screening process.

### Eligibility criteria and study selection

Our article eligibility screening process primarily focused on randomized controlled trials (RCTs) and non-randomized comparative studies. Initially, we assessed titles and abstracts during the screening phase, followed by a thorough examination of the selected study texts. The included studies specifically investigated the effects of various anesthetic drugs (such as ketamine or propofol and others) in upper gastrointestinal endoscopy procedures among school-aged children (aged 6 to 12 years old). The primary objective was to directly compare the clinical effectiveness of these interventions, which encompassed factors such as length of stay in the Post Anesthesia Care Unit (PACU), endoscopist satisfaction, heart rate, mean arterial pressure, recovery time, and adverse reactions. Adverse reactions assessed during induction and the procedure included hypoxemia, hypotension, bradycardia, tachycardia, coughing, and hiccups, while adverse reactions during recovery encompassed symptoms such as headache, dizziness, vomiting, nausea, visual disturbances, and hallucinations. Any discrepancies were addressed by consulting the primary author for resolution.

### Exclusion criteria

We omitted prospective cohort studies, retrospective cohort studies, case–control studies, case series, case reports, editorials, cross-sectional studies, and studies involving non-human subjects from our analysis. Additionally, we excluded studies examining anesthetic treatments in patients outside the age range of school-aged children (6 to 12 years old), as well as non-English studies and those with unreliable data.

### Quality assessment

We utilized the Cochrane Collaboration tool for assessing the risk of bias in RCTs (ROB-2) (19)to evaluate the quality of the studies included. In this tool, each RCT undergoes assessment for potential risks, including (1) selection bias, which involves evaluating methods for random sequence generation and allocation concealment; (2) performance bias, assessed by examining blinding procedures for participants and study personnel; (3) detection bias, evaluated through blinding methods applied to outcome assessment; (4) attrition bias, which includes assessing the extent and impact of incomplete outcome data and the appropriateness of statistical analysis techniques used to address it; (5) reporting bias, determined by assessing the consistency of reported outcomes with the pre-specified methods outlined in clinical trial registration; and (6) any other potential sources of bias that could have influenced the study data. Discrepancies were addressed through discussions between two team members.

### Data extraction and study outcomes

We adopted a standardized method for data extraction utilizing a pre-defined Excel sheet, which included details regarding study characteristics, patient demographics, and outcomes. Any discrepancies were resolved through discussion or consultation with the primary author. Relevant information encompassing study features, patient profiles, and safety and efficacy measures was systematically recorded in the designated Excel sheet. In cases where studies reported outcomes across multiple time points, data extraction was performed separately for each time point to facilitate subsequent subgroup analysis.

### Outcome definition

This study rigorously assessed treatment effectiveness and safety, employing a comprehensive set of metrics such as time to Recovery, measured using either the Steward Recovery score of 7 points or the Rapid Evaluation Assessment of Clinical Reasoning Tool (REACT) score of 10 points. Parameters evaluated included hypoxemia (SpO2 < 90% for more than 1 min), hypotension (mean arterial pressure lower than 20% of pre-induction levels), tachycardia (defined as 30% above the average heart rate by age), bradycardia (heart rate less than 60 beats per minute), visual disturbance (blurred or double vision), and endoscopist satisfaction, assessed using a ten-point scale (1–3: unsatisfactory; 4–6: average satisfaction; 7–10: satisfactory). These assessments were conducted from treatment initiation to specified follow-up endpoints.

### GRADE

The certainty of evidence was evaluated using the GRADE (Grading of Recommendations, Assessment, Development, and Evaluation) framework. This assessment considered the following domains: risk of bias, inconsistency, indirectness, imprecision, and other relevant factors. The assessment was performed for the following outcomes: (1) complications during the procedure, (2) complications after the procedure, and (3) heart rate during the procedure and recovery time.

## Results

### Study demographics, characteristics, and quality assessment

**Fig. 1:**
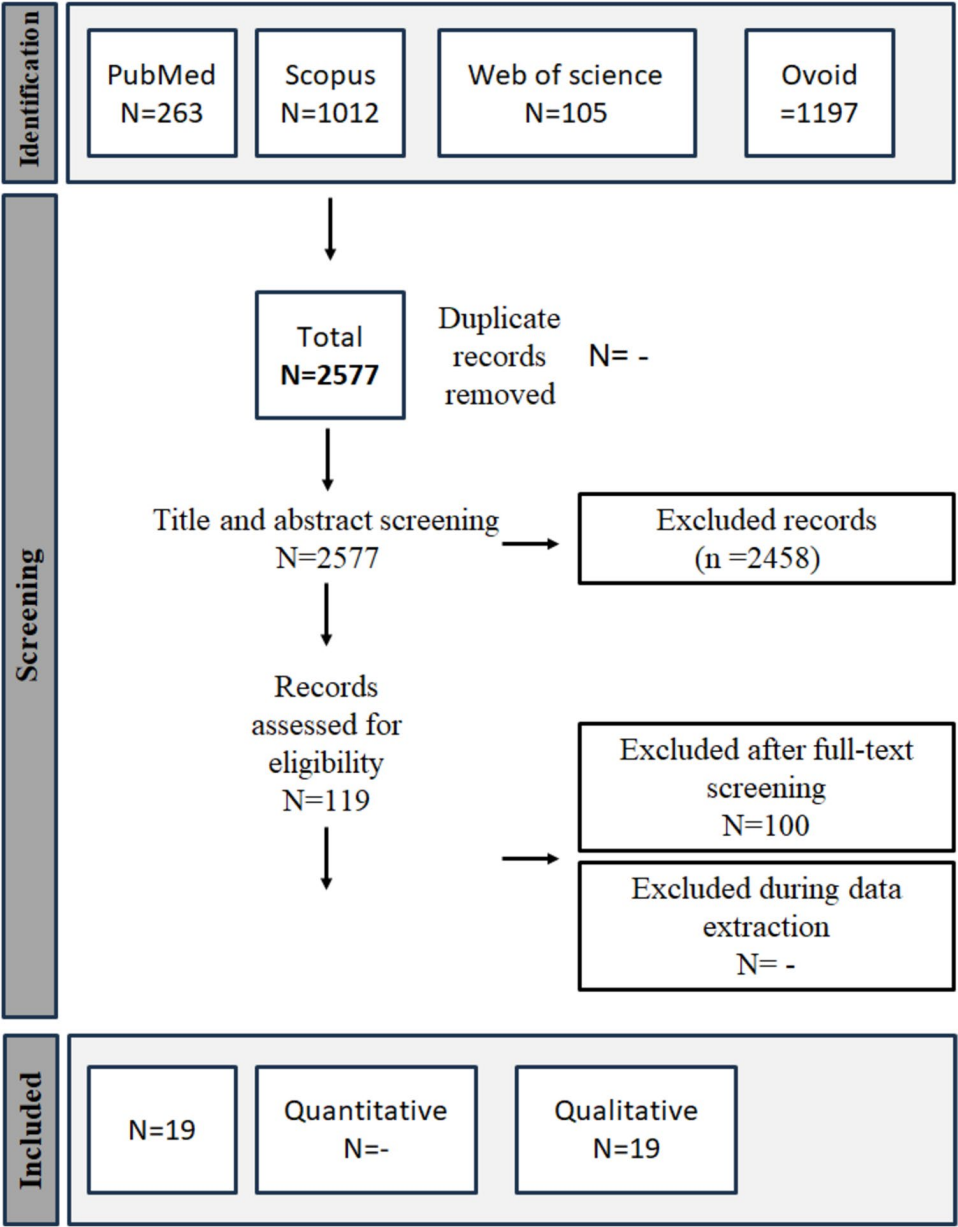
PRISMA flow diagram of the study selection process

**Fig. 2:**
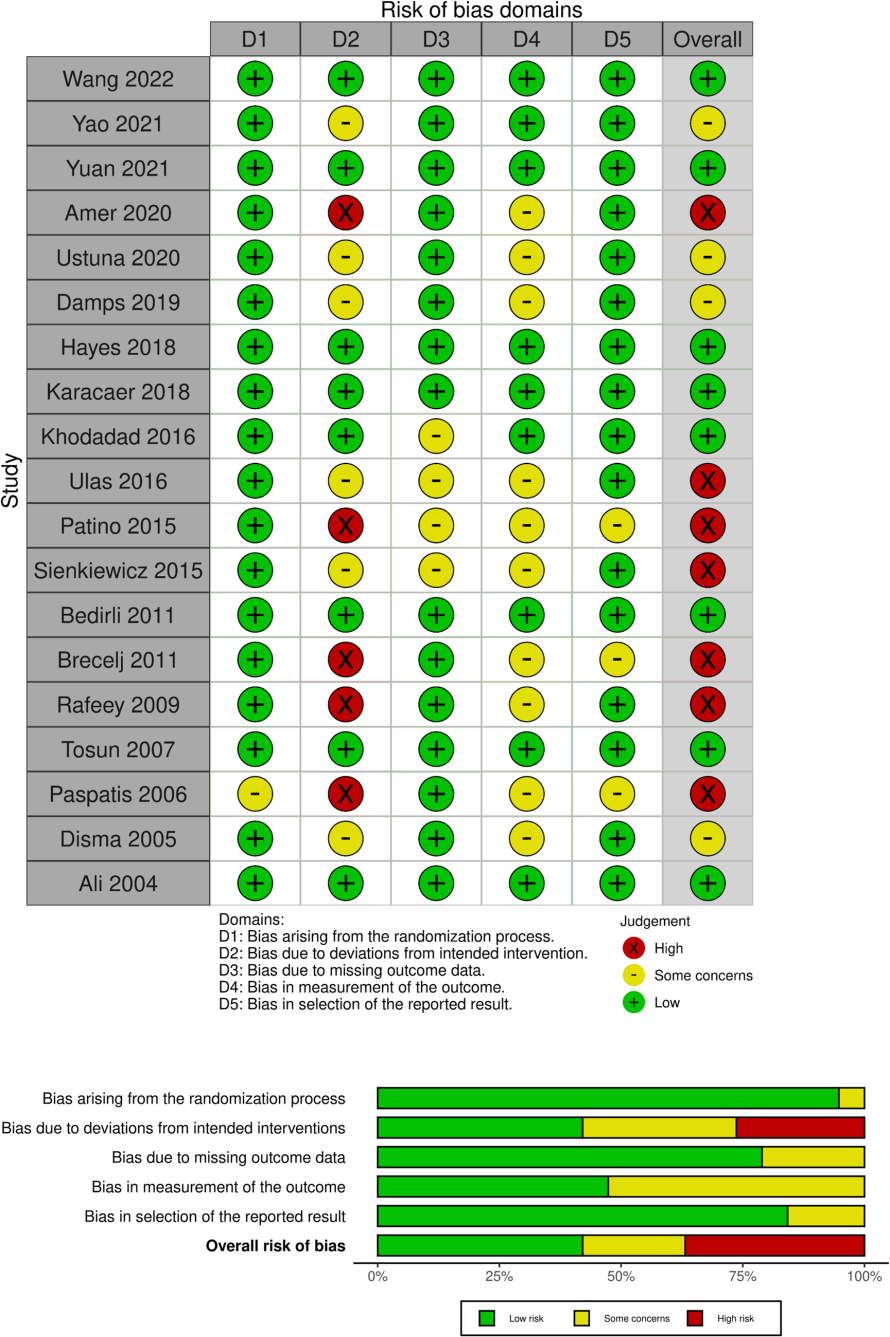
Risk of bias summary for randomized controlled trials using ROB2

A comprehensive search yielded a total of 2,548 studies. Title and abstract screening were started without duplicate detection, and a total of 119 studies were included in full-text screening Fig. 1. Ultimately, 19 randomized controlled trials met our criteria (Hu Z et al., 2022; Amer et al., 2020; Wang et al., 2022; Yao et al., 2021; Damps et al., 2019; Hayes et al 2018; Karacaer 2018; Akbulut et al. 2017; Patino 2015; Sienkiewicz 2015; Bedirli 2012; Brecelj 2012; Rafeey 2010; Tosun 2007; Paspatis 2006; Disma 2005; Ali 2004; Dost 2021; Khodadad 2016; Ustun 2021; and Yuan 2022). The summary and characteristics of the included studies are summarized in Tables 1 and 2, respectively. Our 19 included studies, eight of them were assigned as low risk of bias (Wang et al., 2022; Hayes et al 2018; Karacaer 2018; Bedirli 2012; Tosun 2007; Ali 2004; Khodadad 2016; and Yuan 2022), seven as high risk of bias(Amer et al., 2020; Akbulut et al. 2017; Patino 2015; Sienkiewicz 2015; Brecelj 2012; Rafeey 2010; and Paspatis 2006), and four as some concerns(Yao et al., 2021; Damps et al., 2019; Disma 2005; and Ustun 2021). Detailed description of ROB-2 in Fig. 2.

**Table 1:** Baseline characteristics of the included studies: *ASA* American Society of Anesthesiologists Score, *N/E* not evaluated

Study ID	Groups	Age (years) Mean (SD)		Gender (male) N. (%)		Body height (cm) mean (SD)		Body weight (kg) mean (SD)		Duration of endoscopy (min) mean (SD)		ASA^*^, I, n (%)		ASA, II, n (%)		BMI (kg/m) mean (SD)	
TOSUN 2007	PK (propofol/ketamine)	9.7	4.9	22	47.80%	N/E		N/E		10.9	3.1	N/E		N/E		N/E	
	PF (propofol/fentanyl)	11.25	3.93	24	54.50%					10.7	2						
Akbulut et al. 2017	Midozolam/ketamine	12.21	3.38	N/E		N/E		41.92	15.24	4.55	0.75	N/E		N/E		N/E	
	Fentanyl/propofol																
Patino 2015	IS (intubation with sevoflurane)	7.3	4.56	33	60	N/E		25.57	15.12	N/E		13	60	47	60	16.27	2.28
	IP (intubation with propofol)	6.3	3.8	33	58			24.1	10.64			15	58	43	58	16.6	2.74
	NA (native airway with propofol)	6.67	3.8	35	61			24.8	12.91			10	61	50	61	16.1	2.1
Ustun 2021	Ketamine/propofol	N/E	N/E	N/E	N/E	N/E	N/E	N/E	N/E	N/E	N/E	N/E	N/E	N/E	N/E	N/E	N/E
	Tramadol/propofol	N/E	N/E	N/E	N/E	N/E	N/E	N/E	N/E	N/E	N/E	N/E	N/E	N/E	N/E	N/E	N/E
Wang 2022	Group P (S-ketamine 0 mg/kg)	9.41	2.06	13	43.30%	139.97	15.89	34.7	12.1	13.97	3.38	N/E	N/E	N/E	N/E	17.1	2.8
	Group S0.3 (S-ketamine 0.3 mg/kg)	9.92	1.87	17	58.60%	138.62	11.58	35.72	10.85	12.55	3.86	N/E	N/E	N/E	N/E	18.13	3.5
	Group S0.5 (S-ketamine 0.5 mg/kg)	8.93	1.95	17	56.70%	137.47	14.1	34.12	10.32	12.47	2.32	N/E	N/E	N/E	N/E	17.4	3.02
	Group S0.7 (S-ketamine 0.7 mg/kg)	9.45	1.66	16	53.30%	137.67	8.75	35.52	10.21	13.07	3.48	N/E	N/E	N/E	N/E	18.37	3.75
Yao 2021	Control group (saline)	7.1	1.9	15	75%	122	11.1	23.2	5.2	25.9	7	19	95%	1	5%	15.4	1.2
	Lidocaine (1.5 mg/kg)	7.5	2.1	12	60%	124.7	12.4	24.9	5.7	24	5.8	18	90%	2	10%	15.8	1.1
Yuan 2022	Group S (saline)	6.5	2.4	11	55%	1.16	0.14	24.8	5.68	12.4	1.67	N/E	N/E	N/E	N/E	21.25	3.06
	Group L (lidocaine)	7	3.2	8	40%	1.15	0.14	25.2	7.36	12.35	1.81	N/E	N/E	N/E	N/E	21.59	4.27
Amer 2020	Dexmedetomidine-ketamine	3.5	1.6	30	50%	N/E	N/E	15	4	5.7	2.2	48	80%	12	20%	N/E	N/E
	Propofol-ketamine	4.25	1.7	24	40%	N/E	N/E	17.3	5.6	5.6	1.9	42	70%	18	30%	N/E	N/E
Brecelj 2012	Midazolam and ketamine	8.9	4.5	57	59%	N/E	N/E	32.6	18.5	N/E	N/E	N/E	N/E	N/E	N/E	N/E	N/E
	Ketamine	8.8	4.3	54	52%	N/E	N/E	37.2	13.9	N/E	N/E	N/E	N/E	N/E	N/E	N/E	N/E
Damps 2019	Propofol and ketamine	13	9	18	39.10%	N/E	N/E	N/E	N/E	9	2	46	100%	N/E	N/E	N/E	N/E
	Propofol and remifentanil	12	8	14	31.80%	N/E	N/E	N/E	N/E	9	2	44	100%	N/E	N/E	N/E	N/E
Hayes 2018	Ketamine 0 mg/kg	8.4	(4–12)	N/E		1.3	0.2	30.1	12.4	8.6	2.7	N/E				16.2	2.6
	Ketamine 0.25 mg/kg	8.4	(4–12)			1.3	1.9	28.5	8.9	7.9	1.6					15.5	1.5
	Ketamine 0.5 mg/kg	8.4	(4–12)			1.3	0.15	29.5	10.9	7.5	2.1					19.9	4
	Ketamine 1 mg/kg	8.4	(3–12)			1.3	0.19	30	10.7	6.8	2.1					16.1	3.2
Karacaer 2018	Remifentanil and ketamine	9	4.1	17	50%	N/E		31.3	17.1	18.7	8	70		70		N/E	
	Propofol and ketamine	9.9	4	19	54.20%			35.7	18.8	22.5	9						
Khodadad 2016	0.5 mg/kg oral midazolam	6.8	3.3	58	48.70%	N/E		N/E		N/E		N/E		N/E		N/E	
	0.1 mg/kg IV midazolam																
Sienkiewicz 2015	Midazolam	13.46	1.87	12	48%	161.3	13.96	50.55	12.53	N/E		17	68%	8	32%	N/E	
	Propofol	12.62	1.65	14	53.80%	159.73	13.14	46.35	12.91			22	84.6	4	15.40%		
Bedirli 2012	Fentanyl with propofol	N/E		16	40%	N/E		27.2	12.5	15.5	5.4	N/E		N/E		N/E	
	Tramadol with propofol	N/E		22	55%	N/E		24.3	11.5	15.5	5.4	N/E		N/E		N/E	
Rafeey 2010	Oral midazolam	6.3	2.91	23	37.70%	N/E		N/E		23.9	13.1	N/E		N/E		N/E	
	I.V midazolam	7.98	3.71			N/E		N/E		25.5	11.3	N/E		N/E		N/E	
ALI 2004	Fentanyl	9.5	3.9	6	54.55%	N/E		N/E		13.2	8.8	N/E		N/E		N/E	
	Meperidine	11.2	4.8	5	38.46%	N/E		N/E		12.8	8.1	N/E		N/E		N/E	
Disma 2005	Propofol alone (Group P)	6.7	2.9	46	57.50%	N/E		22.7	10.8	7.5	2.5	N/E		N/E		N/E	
	Propofol with midazolam 0.1 mg kg^−1^(Group PM)	7.1	3.1	38	48.72%	N/E		27.5	16.2	7.3	2.5	N/E		N/E		N/E	
	propofol with fentanyl 1 μg kg^−1^(Group PF)	6.8	2.8	39	47.56%	N/E		25.6	9	6.9	2.5	N/E		N/E		N/E	
Paspatis 2006	Oral midazolam as a premedication and intravenous propofol Group A	8.1	2.9	13	50.00%	N/E		31.6	11.1	10.1	4.2	24	92.30%	2	7.70%	N/E	
	Intravenous propofol alone Group B	9	3.3	13	46.40%	N/E		35.4	13.3	10	5.5	26	92.80%	2	7.20%	N/E

**Table 2:** Summary of the included studies

STUDY	STUDY DESIGN	COUNTRY	ANESTHESIA INDUCTION				ANESTHESIA MAINTENANCE		TOTAL PARTICIPANTS	FOLLOW-UP DURATION	MAIN INCLUSION CITERIA	PRIMARY OUTCOMES	CONCLUSION
TOSUN^2007^	prospective, randomized, double blinded	Turkey	Propofol/Ketamen	Propofol/fentanyl			propofol		90	2 hours	ASA I-II, aged 1 to 16-year-old patients	"Heart rate (HR), systolic arterial pressure (SAP), peripheral oxygen saturation (SpO2), respiratory rate (RR), and Ramsey sedation scores"	the PK combination provided better tolerance of endoscope insertion and better hemodynamic stability but side effects were more frequent in this group. Restlessness during endoscopy was observed more often in the PF group than in the PK group.
AKBULUT et al. 2017)	prospective, randomized, single blinded	Turkey	Midozolam/ketamine	Fentanyl/propofol			Ketamine	propofol	238	N/E	patients who underwent UGE for diagnostic purposes	RSS	-midazolam–ketamine combination were more comfortable than that in the fentanyl–propofol group during the procedure. -the recovery time was longer in the midazolam–ketamine group. -the fentanyl–propofol group was more comfortable in the recovery period in terms of complications."
PATINO 2015	prospective, randomized	USA	Group IS (Sevoflurane)	Group IP-airway management with intubation and anesthetic maintenance with propofol	Group NA-native airway with oxygen supplementation by nasal cannula and maintenance with propofol.		N/E		180	N/E	children aged 1–12 years with ASA physical status I or II presenting for outpatient EGD	-incidence of respiratory complications: minor desaturation (SpO2 between 94% and 85%), severe desaturation (SpO2 < 85%), apnea, bronchospasm, aspiration, airway obstruction, laryngospasm, and trauma during airway manipulation.	-an increased incidence of respiratory complications in nonintubated patients maintained with propofol; endotracheal intubation was seen to greatly reduce the incidence of adverse airway events during EGD.-the general anesthesia with propofol and the native airway during EGD was associated with a higher incidence of respiratory complications, including minor desaturation, severe desaturation, inadequate anesthesia and apnea, compared with endotracheal intubation; no improvement in institutional efficiency was seen with avoidance of endotracheal intubation.
USTUN 2021	prospective, randomized	Turkey	Ketamine/propofol	Tramadol/propofol			Propofol		80	N/E	ASA physical status I–II patients, ages 1 to 18 who were scheduled for upper gastrointestinal endoscopies (UGIE)	The heart rate (HR), mean arterial pressure (MAP), peripheral oxygen saturation (SpO2), respiratory rate (RR), and Ramsay sedation scores	combination of tramadol-propofol result in a faster recovery without increase the rate of adverse effects. However, patients required additional propofol due to insufficient sedation, so tramadol should be considered for short procedures.
WANG 2022	prospective, randomized, double-blinded	China	Group P (S-Ketamine 0 mg/kg)	Group S0.3 (S-Ketamine 0.3 mg/kg)	Group S0.5 (S-Ketamine 0.5 mg/kg)	Group S0.7 (S-Ketamine 0.7 mg/kg)	Propofol		120	N/E	School-aged children (6 to 12 years old) with ASA I or II and undergoing gastro duodenoscopy were enrolled.	smooth placement rate of first endoscope insertion.	-S-ketamine could improve the tolerance and the smooth placement rate during endoscope insertion, which was positively related to the dosage of S-ketamine.-combined administration of S-ketamine and propofol can increase the tolerance of school- aged children during endoscopic insertion. Moreover, the smooth placement rate during the frst endoscope insertion is positively correlated with the dose of S-ket- amine. S-ketamine administration at 0.7 mg.kg−1can maintain hemodynamic stability in children, reduce the number of additional propofol and the total amount of propofol, and improve endoscopist satisfaction. However, dizziness is the most common adverse event with 73.3% incidence and may prolong PACU stay.
YAO 2021	prospective, randomized	China	Lidocaine (1.5 mg/kg)	Control group (Saline)			lidocaine	saline	40	60 min after the procedure	"children aged 5–12 years who were scheduled for colonoscopy"	propofol requirement,	intravenous lidocaine can be safely used in paediatric patients undergoing colonoscopy, reducing the required propofol by 35.5%. Awakening and recovery times were signifcantly reduced in the lidocaine group, as well as the number of involuntary body movements. Patients in the lidocaine group had signifcantly lower pain scores after colonoscopy than those in the control group.
YUAN 2022	prospective, randomized, double-blinded, placebo	China	Group S (Saline)	Group L (Lidocaine)			Sufentanil	Propofol	40	N/E	children aged from 3 to 10 years undergoing colonoscopy under sedation were included in our study,	Intraoperative propofol and sufentanil requirements and the number of oxygen desaturation episodes (defined as peripheral capillary oxygen desaturation (SpO2) less than 95% and 90%).	adding i.v. lidocaine as an adjuvant drug could significantly reduce the propofol and sufentanil requirements for pediatric colonoscopy and at the same time, shorten the recovery time.
AMER 2020	RCT	Egypt	Dexmedetomidine-ketamine	Propofol-ketamine					120	N/E	Patients with the ASA physical status I-II, between 2 and 7 years old who are scheduled for elective diagnostic upper gastrointestinal endoscopy	Hemodynamic variables including Heart Rate (HR), Mean Arterial Pressure (MAP), Respiratory Rate (RR) and Oxygen Saturation (SpO2), The incidence of significant hypotension, recovery time, dosage of anesthesia, post-procedure complications, and endoscopist satisfaction	Propofol-ketamine was superior in reducing recovery time for pediatric patients undergoing upper gastrointestinal endoscopy and is prefereable is short diagnostic procedures while Dexmedetomidine-ketamine required a lower dose so it is better in long procedures
BRECELJ 2012	RCT	Slovenia	Midazolam and Ketamine	Ketamine			Propofol		201	atleast 1 month	Patients between 1-19 years old suitable for IV anesthesia	Adverse events	The addition of midazolam to ketamine sedation proved to be safe with the ability to reduce the number of emergency events in the hospital
DAMPS 2019	RCT	Poland	Propofol and Ketamine	Propofol and Remifentanil			Propofol	Propofol and Remifentanil	90	N/E	Children with ASA I who were enrolled for elective endoscopy of the upper gastrointestinal tract under general anaesthesia	respiratory and circulatory parameters, adverse events, waking time and the child’s condition post-operation	The addition of ketamine to propofol was superior in induction of anesthesia while the addition of remifentanil was superior in recovery and improving post-operative mood and both additions were safe
HAYES 2018	Randomized, double-blind, four-arm trial	Canada	Ketamine 0 mg/kg	Ketamine 0.25 mg/kg	Ketamine 0.5 mg/kg	Ketamine 1 mg/kg	Propofol		56	N/E	Children aged 3-12 yr undergoing elective gastro-duodenoscopy	- ED50 of propofol - Mean arterial pressure - Incidence of nausea and visual disturbances	Ketamine at 0.5e1 mg kge1 reduces the dose of propofol required to provide general anaesthesia for gastro-duodenoscopy in children and may reduce the incidence of propofol-related changes in haemodynamics.
KARACAER 2018	Randomised, double blind	Turkey	Remifentanil and ketamine	Propofol and Ketamine			Remifentanil	Propofol	70	N/E	ASA I-II Children aged 2-16-years undergoing colonoscopy procedure with sedation	- To measure the efficacy of PK and RK combinations on RSSs	Coadministration of ketamine with either remifentanil or propofol effectively and safely provides sedation and analgesia in children undergoing colonoscopy. Sedation scores were significantly better in remifentanil-ketamine group than in propofol-ketamine group
KHODADAD 2016	Randomised, double blind	Iran	0.5 mg/kg Oral midazolam	0.1 mg/kg IV midazolam			N/E		120	N/E	1 to 16-year-old patients who require diagnostic upper endoscopy and the ASA class I and II.	- The assessment of the safety and efficacy of oral midazolam and intravenous midazolam in terms of vital signs, oxygen saturation, and adverse effects.	Oral or IV midazolam were not able to put most patients in deep sedation level
SIENKIEWICSZ 2015	Blind randomized trial	Poland	atropine (10 mcg/kg)	alfentanyl (5 mcg/kg)	lignocaine (0.5 mg/kg)	midazolam (0.025 mg/kg).	propofol	Midazolam	51	120 minutes after the procedure	Children at the age of 9–16 years, classified to the first or second class of the American Society of Anaesthesiologists’ physical status classification	level of pain, level of pain, Procedure memory	In children sedated for EGD, propofol is significantly better than midazolam at providing procedural amnesia and controlling behaviour during the procedure.
BEDIRLI 2012	Randomised, double blind	Turkey	fentanyl (2 ug.Kg-1) with propofol (1.5 ug.Kg-1)	Tramadol (2 ug.Kg-1) with propofol (1.5 ug.Kg-1)			N/E		80	5 minutes, 15 minutes, 20 minutes	patients with ASA I-II, aged 1–16 years,	changes in oxygen saturation, changes in sedation score, Adverse effects	Tramadol in pediatric patients undergoing UGIE provided sedation as efficient as fentanyl with a better hemodynamic and respiratory stability and provided a superior safety and tolerance in younger children.
RAFEEY 2010	prospective, randomized	Iran	oral midazolam (0.5 mg/kg)	i.v. midazolam (0.05–0.1 mg/kg)			N/E		61	SpO2, HR, SAP, and RR were recorded just before the midazolam was introduced (T0m), 10 min after midazolam (T10m), during the procedure (Ten), and 10 min after removal of the endoscope (Ten10).	61 consecutive children who underwent upper gastrointestinal endoscopy were prospectively included in the study (range, 1–16 years)	compare the safety and efficacy of oral versus i.v. midazolam in providing sedation for pediatric upper gastrointestinal (GI) endoscopy	Oral administration of midazolam is a safe and effective method of sedation that significantly reduces anxiety and improves overall tolerance for children undergoing esophagogastroduodenoscopy.
ALI 2004	double-blind, randomized trial	USA	fentanyl (1 ¹g/kg)	meperidine (1 mg/kg)			midazolam		24	60 and 120 min after the procedure	Pediatric patients between 2 and 18 years of age who underwent esophagogastrodu odenoscopy (EGD) and/or colonoscopy for routine clinical indications	This study compared the safety and efficacy of fentanyl and meperidine for analgesia in pediatric gastrointestinal endoscopy	Meperidine and Fentanyl are equally effective in providing analgesia for pediatric gastrointestinal endoscopy.
DISMA 2005	randomized, prospective, three-study-group	Italy	propofol alone (Group P)	propofol with fentanyl 1 μg kg 1 (Group PF)	propofol with midazolam 0.1mgkg 1 (Group PM)		Additional doses of propofol		240	Patient recovery was assessed at 5 min intervals	paediatric outpatients, ASA I–II, aged 1–12-yr old, scheduled for diagnostic endoscopic procedures of the upper gastrointestinal tract	investigate sedation in children using propofol alone or combined with fentanyl or midazolam with regard to efficacy, adverse reactions or side-effects related to the drugs, ease of operation for the endoscopist, and time to discharge from the post-anaesthesia care unit	Propofol in combination with fentanyl or midazolam gives better sedation and ease of endoscopy than propofol alone
PASPATIS 2006	Prospective, Randomized Study	Greece	oral 0.5 mg/kg of midazolam (maximum dose 20 mg) and IV doses of propofol 0.5 mg/kg	IV doses of propofol 0.5 mg/kg			N/E		54	N/E	Children (aged 3 y or older) who underwent Upper Gastrointestinal Endoscopies (UGIE)	compare the required dose of intravenous (IV) propofol between group A (synergistic sedation with an oral dose of midazolam combined with IV propofol) and group B (IV propofol alone), in diagnostic upper gastrointestinal endoscopy (UGIE) in pediatric patients	Synergistic sedation with an oral dose of midazolam combined with propofol may benefit the children who undergo UGIE with regard to lower mean dose of propofol used, easier IV line placement, easier separation from the parents, less pain induced by the IV line placement and greater patient comfort

*N/E* not evaluated

### Summary of study findings

#### Complications during the procedure

Respiratory complications demonstrated significant variability across regimens. Hypoxia incidence was highest in fentanyl-based combinations, affecting 8.9% (4/45) of propofol/ketamine (PK) group patients and 4.2% (5/119) of propofol/fentanyl (PF) recipients, compared to 6.8% (3/44) in PK and 2.9% (1/34) in remifentanil/ketamine (RK) groups. Apnea showed particularly high rates in natural airway propofol administration (8.6%, 5/58), exceeding propofol alone (2.5%, 2/80) and PF (2.4%, 2/82). Cough prevalence reached 7.5% (6/80) with propofol monotherapy and 5.0% (6/119) with midazolam/ketamine (MK), while maintaining consistent 12–15% rates across S-ketamine dose groups. Cardiovascular effects revealed bradycardia in PF 2.2% (1/45) and 4.5% (2/44) in PK groups. Tachycardia affected 4.2% (5/119) of PF and 10.0% (4/40) of PK recipients. Gastrointestinal disturbances showed nausea/vomiting rates of 27.3% (12/44) in PK, 15.5% (9/58) in propofol intubation, and 6.7% (4/60) in dexmedetomidine/ketamine (DK) groups, while hypersalivation affected 16.8% (20/119) of MK and 26.9% (32/119) of PF patients. Neurological effects included striking dizziness rates of 34.1% (15/44) in PK groups, escalating from 42.9% (6/14) to 64.3% (9/14) with ketamine dose increases, while visual disturbances were exclusive to PK at 18.2% (8/44). Safety profiles showed PK and midazolam monotherapy maintained 0% adverse event rates, whereas propofol/tramadol (PT) demonstrated favorable profiles with only 2.5% (1/40) bradycardia and 7.5% (3/40) vomiting incidence. This quantitative analysis reveals ketamine’s neurological effects (34.1% dizziness), opioids’ respiratory risks (8.9% hypoxia), and propofol’s airway complications (7.5% cough), providing evidence-based guidance for regimen selection. Detailed complications are provided in ESM. 3.

#### Complications after the procedure

The reviewed studies demonstrated variable post-sedation complication profiles. Most regimens showed good tolerability, with Tosun et al. (2007), Ustun et al. (2021), and Paspatis et al. (2006) reporting no adverse events in their respective PK, PT, and propofol/midazolam (PM) groups. However, Akbulut et al. (2017) documented significant neuropsychiatric effects in the PF group, including visual disturbances in 73.1% (87/119) of patients and hallucinations in 15.1% (18/119), compared to 8.4% (10/119) and 1.7% (2/119) respectively in the MK group. Gastrointestinal effects showed vomiting in 33.6% (40/119) of PF recipients versus 1.7% (2/119) with MK.

Dose-dependent neurological effects emerged clearly in Wang’s et al. (2022) S-ketamine study, where dizziness prevalence escalated from 26.7% (8/30) in the placebo group to 40.0% (12/30) at 0.3 mg/kg, 43.3% (13/30) at 0.5 mg/kg, and 73.3% (22/30) at 0.7 mg/kg. Visual disturbances showed a non-linear dose relationship, peaking at 27.6% (8/29) in the 0.3 mg/kg group. Headache incidence remained low (≤ 10%) across all doses.

#### Heart rate during the procedure and recovery time

The analysis of procedural and recovery outcomes across 14 sedation studies revealed several key findings regarding hemodynamic stability and recovery profiles. The comparative study by Tosun et al. (2007) demonstrated that PK combination better maintained heart rate stability (100 ± 17.6 bpm baseline to 92.2 ± 16.8 bpm at 10 min) compared to PF, which showed more pronounced bradycardia (101 ± 17.6 bpm to 76.8 ± 13.8 bpm), suggesting ketamine’s protective effect against propofol-induced cardiovascular depression. Wang et al. (2022) established a dose-dependent relationship with S-ketamine, where the 0.5 mg/kg dose produced optimal heart rate maintenance (93.81 bpm) but paradoxically required the longest recovery time (35.67 min), while higher 0.7 mg/kg doses showed faster recovery (33.5 min), potentially due to bimodal pharmacokinetic effects.

Recovery characteristics varied significantly between different sedation approaches. The fastest recovery profiles were observed with lidocaine adjuncts (18–22 min PACU stays in Yao et al., 2021; Yuan, et al., 2022) studies) and high-dose S-ketamine regimens (33.5 min), while DK combinations demonstrated the most prolonged recovery (42 ± 6 min in Amer et al., (Amer, et al., 2020). Midazolam-based sedation showed consistent intermediate recovery times (28–32 min in Rafeey 2010), and all propofol-based regimens, regardless of adjuncts, required extended PACU stays exceeding 50 min (Disma 2005). These findings highlight important clinical trade-offs—while ketamine combinations provide superior hemodynamic stability, they may prolong recovery, whereas lidocaine adjuncts offer the most efficient recovery but may require additional agents for adequate procedural sedation. The data suggest that sedation protocol selection should be individualized based on procedure duration, patient cardiovascular risk factors, and facility recovery capacity. A summary of the included study results is in ESM. 2.

#### GRADE

The certainty of evidence, evaluated using the GRADE framework, was moderate to low for most outcomes due to methodological limitations (Guyatt et al., 2008). For complications during the procedure, the evidence was downgraded twice due to serious risk of bias (7 out of 19 studies had high bias) and inconsistency, as complication rates varied significantly (e.g., hypoxia ranged from 2.9 to 8.9%). Similarly, for complications after the procedure, the certainty was reduced due to risk of bias and imprecision, as many studies had small sample sizes (40–119 patients per group) and wide confidence intervals, as shown in ESM. 4.

## Discussion

Due to the nature of the upper gastrointestinal endoscopy, it is important to apply anesthetics before the procedure to prevent anxiety and agitation during the procedure, to enhance the success of the procedure, especially in children (Oliva S et al., 2017; Isoldi et al., 2021; Lichtenstein et al., 2008; and Lin et al., 2017). Until now, different regimens of sedation formulas have been applied, and no formula has been accepted universally (Rana et al., 2016). The combination of short-acting hypnotics and analgesics is the most commonly used regimen (Rana et al., 2016; Childers et al., 2015). Propofol, a hypnotic agent, is commonly used for its rapid and effective action (Early DS et al., 2018). However, it was linked to cardiovascular and respiratory depression (10,11). The addition of analgesics to the sedative agents is to increase their potency and reduce pain (Lichtenstein et al., 2008; Early DS et al., 2018). While the addition of opioids to propofol proved their efficiency, it led to an exacerbation of the depression of the cardiovascular and respiratory systems (Shetabi et al., 2018). While opioids are the most commonly used analgesic, other alternatives have shown superiority over opioids, such as ketamine and dexmedetomidine (Hu et al., 2022; Amer et al., 2020). In this review, we conducted a focused literature search for articles assessing the efficacy and safety of different anesthetics applied to children undergoing upper gastrointestinal endoscopy.

### Complications after the procedure

No complications after the procedure were observed with the use of propofol alone or with other drugs like ketamine, fentanyl, midazolam, or tramadol. However, it was associated with the incidence of nausea and vomiting during the procedure. Also, there was a high reduction in the heart rate after 10 min of induction in the propofol combined with fentanyl compared to the propofol combined with ketamine. Cardiorespiratory depression was frequently reported with using ketamine alone.

### Complications during the procedure

Apnea was frequently reported with the use of midazolam, ketamine, and propofol. In Wang et al. (2022), group S0.5 (S-ketamine 0.5 mg/kg) showed the lowest reduction in heart rate compared to other applied doses in the study. The use of ketamine combined with propofol led to a higher reduction in the heart rate compared to ketamine alone. Ketamine was the most reported anesthetic to cause nausea and vomiting. Ketamine was also associated with the highest incidence of cough and hypotension. The application of DK was associated with lower heart rates and longer discharge times compared to PK.

Disma et al. (2005) showed a similar PACU stay with the use of propofol alone or combined with midazolam or fentanyl. Tachycardia was observed with the use of propofol in Ustun et al. (2021) and Disma et al. (2005), which might be caused by a reflex to hypotension. The highest incidence of laryngeal spasms and hypoxia was observed with the use of propofol. Also, propofol was the only anesthetic to cause visual disturbances.

Lidocaine was linked to shortened PACU and an increased incidence of hypoxia, bradycardia, and hypotension.

While our analysis found midazolam (oral or IV) to be a safe option with no reported adverse events and comparable recovery times between routes, several limitations from broader evidence warrant consideration. First, midazolam alone may not reliably achieve deep sedation, particularly for stimulating procedures like upper endoscopy. Unlike ketamine or propofol-based regimens, its sedative effects are dose-dependent and may be insufficient for some patients, potentially compromising procedural conditions or requiring rescue dosing, though this was not observed in our cohort (Karacaer et al., 2018). Second, while our data showed no respiratory events with midazolam, previous systematic reviews note that higher doses (e.g., > 0.1 mg/kg IV) can cause dose-related respiratory depression, especially when combined with opioids, contrasting with the hypoxia rates seen in PF (8.9%) and apnea in propofol monotherapy (8.6%) in our analysis (Vet et al., 2016). Third, recovery times for midazolam were similar between oral and IV routes in our study, but other trials report prolonged recovery compared to propofol or ketamine combinations (e.g., DK 42 ± 6 min vs. midazolam 28–32 min), possibly due to its active metabolite (α-hydroxymidazolam) in children with hepatic impairment (Gottschling S et al., 2005). Additionally, midazolam provides no intrinsic analgesia, unlike ketamine or opioids, which may explain the higher cough rates (5.0%) in MK groups, suggesting ketamine’s added benefit for painful stimuli(Krauss and Green, 2000). Finally, neuropsychiatric effects, though absent in our results, are another concern, as midazolam has been linked to paradoxical reactions (agitation, disinhibition) in 1–15% of pediatric patients, particularly those with developmental disorders—a risk not seen with propofol or dexmedetomidine (Krauss and Green, 2000).

### Strengths and limitations

Our study has important strength points such as the following: (1) this study comprehensively reviewed the application of different anesthetic regimens in children; (2) we included the randomized controlled trials only; (3) the demographic data were similar across the included studies; (4) and the overall risk of bias of the included studies was low. However, this study has several limitations: (1) different follow-up periods, (2) no details about the sequence generation during the randomization process, (3) some studies have shown some concerns about the allocation concealment during the randomization process, (4) no studies assessed the least required regimen that can suppress the stress during the insertion of the endoscopy in children, and (5) the hypoxia in the included studies needs to be timely monitored.

### Conclusion

We comprehensively reviewed randomized controlled trials evaluating anesthetic regimens for children undergoing upper gastrointestinal endoscopy. Pooled adverse event trends reveal distinct risk profiles: respiratory complications were most frequent with fentanyl-based combinations (hypoxia 8.9% with PF vs. 2.9% with RK), while propofol monotherapy carried a higher cough risk (7.5%). Cardiovascular instability was prominent with PF (bradycardia 24.4% vs. ≤ 4.5% in ketamine combinations), whereas PK better maintained heart rate stability (92.2 ± 16.8 bpm intraprocedural). Neurological effects dominated ketamine regimens, with dizziness escalating dose-dependently (34.1% overall, peaking at 73.3% with S-ketamine 0.7 mg/kg) and post-procedure hallucinations in 15.1% of PF recipients. Recovery times varied markedly, with lidocaine adjuncts facilitating the fastest recovery (18–22 min) and DK prolonging PACU stays (42 ± 6 min). Midazolam emerged as the safest option (0% adverse events in monotherapy), whereas propofol—alone or combined—consistently linked to cardiorespiratory depression and nausea/vomiting (27.3% with propofol/ketamine). Ketamine, though hemodynamically protective, uniquely caused post-procedure complications (e.g., dose-dependent dizziness, hallucinations). These pooled trends underscore the need for regimen individualization: ketamine combinations for hemodynamic stability (despite longer recovery), lidocaine adjuncts for efficiency, and midazolam for low-risk cases. Larger trials are warranted to define minimal effective doses, balancing safety and sedation depth.

## Supplementary Information

Below is the link to the electronic supplementary material.

ESM1 (DOCX 547 KB)

## Data Availability

The datasets used and/or analyzed during the current study are available from the corresponding author on reasonable request.
