# Supplementary material for: A comprehensive systematic review of randomized controlled trials on anesthetic agents in children’s upper gastrointestinal endoscopy: highlighting safety concerns and efficacy
Source: Naunyn Schmiedebergs Arch Pharmacol. 2025 Sep 4;399(2):1851–69. doi: 10.1007/s00210-025-04557-2 (PMC12901073; doi:10.1007/s00210-025-04557-2)

Contents

[ESM. 1. 2](#_Toc205212248)

[ESM. 2. 3](#_Toc205212249)

[ESM. 3. Detailed adverse events listing 6](#_Toc205212250)

[ESM. 3A. 6](#_Toc205212251)

[ESM. 3B. 7](#_Toc205212252)

[ESM. 4. GRADE assessment of included outcomes 8](#_Toc205212253)

# ESM. 1.

**Search strategy**

| **Database** | **Search strategy** | **Number** |
| --- | --- | --- |
| PubMed | (upper gastrointestinal endoscopy AND children) AND (Propofol OR Ketamine OR Fentanyl OR Midazolam OR Tramadol OR S-Ketamine OR Saline OR Lidocaine OR Dexmedetomidine OR Remifentanil OR Oral Midazolam OR Intravenous Midazolam OR Oral Midazolam as a premedication OR Intravenous Propofol alone OR Meperidine OR Isoflurane OR Sevoflurane OR Nitrous oxide OR Chloral hydrate OR Etomidate OR Thiopental OR Pentobarbital OR Halothane OR Desflurane OR Alfentanil OR Sufentanil OR Remifentanil OR Morphine OR Diazepam OR Lorazepam OR Oxycodone OR Hydromorphone) | 263 |
| Ovoid |  | 1197 |
| Web of science |  | 105 |
| Scopus | (“upper gastrointestinal endoscopy” AND “children”) AND (“Propofol” OR “Ketamine” OR “Fentanyl” OR “Midazolam” OR “Tramadol” OR “S-Ketamine” OR “Saline” OR “Lidocaine” OR “Dexmedetomidine” OR “Remifentanil” OR “Oral Midazolam” OR “Intravenous Midazolam” OR “Oral Midazolam as a premedication” OR “Intravenous Propofol alone” OR “Meperidine” OR “Isoflurane” OR “Sevoflurane” OR “Nitrous oxide” OR “Chloral hydrate” OR “Etomidate” OR “Thiopental” OR “Pentobarbital” OR “Halothane” OR “Desflurane” OR “Alfentanil” OR “Sufentanil” OR “Remifentanil” OR “Morphine” OR “Diazepam” OR “Lorazepam” OR “Oxycodone” OR “Hydromorphone”) | 1012 |

# ESM. 2.

| **Study (Year)** | **Intervention Group** | **Number of Participants** | **Complications During Procedure** | **Complications After Procedure** | **Heart Rate and Recovery Time** |
| --- | --- | --- | --- | --- | --- |
| **Tosun et al. (2007)** | PK (Propofol/Ketamine) | 44 | Hypoxia (3, 6.8%), bradycardia (2, 4.5%), vomiting (7, 15.9%), nausea (5, 11.4%), dizziness (15, 34.1%), cough (8, 18.2%), visual disturbance (8, 18.2%) | No adverse events reported | Baseline HR: 100; Change at 5 min: -6.7; Change at 10 min: -7.8. Recovery: Similar across groups. |
|  | PF (Propofol/Fentanyl) | 45 | Hypoxia (4, 8.9%), bradycardia (1, 2.2%), nausea (5, 11.1%), dizziness (4, 8.9%) | No adverse events reported | Baseline HR: 101; Change at 5 min: -22.7; Change at 10 min: -24.2. |
| **Ulas et al. (2016)** | Midazolam/Ketamine | 119 | Hypoxia (1, 0.8%), bradycardia (1, 0.8%), vomiting (6, 5.0%), cough (6, 5.0%), flushing (8, 6.7%), increased oral secretion (20, 16.8%) | Vomiting (2, 1.7%), visual disturbance (10, 8.4%), headache (23, 19.3%), hallucinations (2, 1.7%) | Not reported. |
|  | Fentanyl/Propofol | 119 | Tachycardia (5, 4.2%), vomiting (2, 1.7%), cough (2, 1.7%), flushing (32, 26.9%), increased oral secretion (32, 26.9%), hypoxia (5, 4.2%), bradycardia (11, 9.2%) | Visual disturbance (87, 73.1%), headache (9, 7.6%), hallucinations (18, 15.1%) | Not reported. |
| **Wang et al. (2022)** | Group P (S-Ketamine 0 mg/kg) | 30 | Cough (5-6, ~18.3%), hypoxia (minor), hypotension (minor) | Vomiting (1, 3.3%), visual disturbance (2, 6.7%), dizziness (8, 26.7%), headache (3, 10.0%) | Baseline HR: N/E; Change at 5 min: -5.82; Change at 10 min: -4.56. Discharge time: ~35.67 min. |
|  | Group S0.3 (S-Ketamine 0.3 mg/kg) | 29 | Cough (5-6, ~19.0%), hypoxia (minor), hypotension (minor) | Dizziness (12, 41.4%), headache (1, 3.4%), visual disturbance (8, 27.6%) | Baseline HR: N/E; Change at 5 min: +1.17; Change at 10 min: -4. |
|  | Group S0.5 (S-Ketamine 0.5 mg/kg) | 30 | Cough (5-6, ~18.3%), hypoxia (minor), hypotension (minor) | Dizziness (13, 43.3%), headache (1, 3.3%), visual disturbance (7, 23.3%) | Baseline HR: 93.81; Change at 5 min: +3.1; Change at 10 min: -1.04. Discharge time: ~33.5 min. |
|  | Group S0.7 (S-Ketamine 0.7 mg/kg) | 30 | Cough (5-6, ~18.3%), hypoxia (minor), hypotension (minor) | Dizziness (22, 73.3%), visual disturbance (5, 16.7%) | Baseline HR: N/E; Change at 5 min: -3.04; Change at 10 min: -3.64. Discharge time: ~35.67 min. |
| **Ustun et al. (2020)** | Ketamine/Propofol | 40 | Tachycardia (4, 10.0%), nausea (4, 10.0%), increased oral secretion (1, 2.5%), hypotension (1, 2.5%), laryngeal spasm (1, 2.5%) | No adverse events reported | Not reported. |
|  | Tramadol/Propofol | 40 | Bradycardia (1, 2.5%) | No adverse events reported | Not reported. |
| **Karacaer et al. (2018)** | Remifentanil/Ketamine | 34 | Nausea/vomiting (2, 5.9%), hypoxia (1, 2.9%), vomiting (2, 5.9%) | No adverse events reported | Not reported. |
|  | Propofol/Ketamine | 34 | No adverse effects | No adverse events reported | Not reported. |
| **Yao et al. (2021)** | Control Group | 20 | Hypoxia, bradycardia, hypotension (few cases) | No adverse events reported | Shorter PACU stays with lidocaine. |
|  | Lidocaine Group | 20 | Hypoxia, bradycardia, hypotension (few cases) | No adverse events reported | Shorter PACU stays with lidocaine. |
| **Hayes et al. (2018)** | Ketamine (0 mg/kg) | 14 | Apnea duration (59 sec), dizziness (6/14, 42.9%) | Dizziness and hypotension reported | Varying reductions in heart rate with ketamine. |
|  | Ketamine (0.25 mg/kg) | 14 | Apnea duration (45 sec), dizziness (7/14, 50.0%) | Dizziness and hypotension reported | Varying reductions in heart rate with ketamine. |
|  | Ketamine (0.5 mg/kg) | 13 | Apnea duration (57 sec), dizziness (8/13, 61.5%) | Dizziness and hypotension reported | Varying reductions in heart rate with ketamine. |
|  | Ketamine (1 mg/kg) | 14 | Apnea duration (39 sec), dizziness (9/14, 64.3%) | Dizziness and hypotension reported | Varying reductions in heart rate with ketamine. |
| **Disma et al. (2005)** | Propofol Alone (Group P) | 80 | Apnea (2, 2.5%), tachycardia (1, 1.3%), laryngeal spasm (3, 3.8%), cough (6, 7.5%) | No adverse events reported | PACU stay: 51.5–54 min. |
|  | Propofol/Midazolam (Group PM) | 78 | Tachycardia (1, 1.3%), cough (2, 2.6%) | No adverse events reported | PACU stay: 51.5–54 min. |
|  | Propofol/Fentanyl (Group PF) | 82 | Apnea (2, 2.4%) | No adverse events reported | PACU stay: 51.5–54 min. |
| **Amer et al. (2020)** | Dexmedetomidine-Ketamine | 60 | Nausea (4, 6.7%) | No adverse events reported | Lower heart rates, longer discharge times compared to propofol-ketamine. |
|  | Propofol-Ketamine | 60 | Nausea (2, 3.3%) | No adverse events reported | Lower heart rates, longer discharge times compared to propofol-ketamine. |
| **Rafeey et al. (2009)** | Oral Midazolam | Not reported | No adverse events reported | No adverse events reported | Similar recovery times with oral and IV midazolam. |
|  | IV Midazolam | Not reported | No adverse events reported | No adverse events reported | Similar recovery times with oral and IV midazolam. |
| **Ali et al. (2004)** | Fentanyl | Not reported | No adverse events reported | No adverse events reported | Not reported. |
|  | Meperidine | Not reported | No adverse events reported | No adverse events reported | Not reported. |

# ESM. 3. Detailed adverse events listing

| ESM. 3A. **Complications during the procedure** | The synthesis of results from the included studies encompasses various complications during procedures across different intervention groups. In the study of Tosun et al., 2007, the PK (Propofol/Ketamine) group with 44 participants showed hypoxia (3 cases), bradycardia (2 cases), vomiting (7 cases), nausea (5 cases), dizziness (15 cases) cough (8 cases) and visual disturbance (8 cases), while the PF (Propofol/fentanyl) group with 45 participants experienced hypoxia (4 cases) and bradycardia (1 case), nausea (5 cases), and dizziness (4 cases), Ulas et al. (2016) with 119 participants noted 1 case each of hypoxia and bradycardia, 6 cases of vomiting and cough by equal, 8 cases of flushing, and 20 cases of increased oral secretion for the midazolam/ketamine group. In the fentanyl/propofol group, the authors found 5 cases of tachycardia, 2 cases of vomiting and cough by equal,32 cases of flushing and increased oral secretions, and 5 cases and 11 cases of hypoxia and bradycardia, respectively. The study by Patino et al. (2015) made a revelation in which vomiting/nausea was reported in the IS (intubation with sevoflurane) group (4 cases) out of 60 participants, IP (intubation with propofol) group (9 cases) out of 58 with 1 case of apnea, and NA (natural airway with propofol) group (7 cases) with 5 cases of apnea. Ustun et al (2020) 4 cases of tachycardia and 4 cases of nausea, increased oral secretions, hypotension, and laryngeal spasm 1 case in each of the Ketamine/propofol group with 40 participants; in addition, there was 1 case of bradycardia only in Tramadol/propofol. In the study by Wang et al. (2022), the adverse effects of S-Ketamine administration during the procedure were evaluated across four treatment groups: Group P (S-Ketamine 0 mg/kg), Group S0.3(S-Ketamine 0.3 mg/kg), Group S0.5 (S-Ketamine 0.5 mg/kg), and Group S0.7 (S-Ketamine 0.7 mg/kg).  Hypoxia, coughing, and hypotension are among the negative outcomes assessed. Besides this, the most noticed adverse effect in all three groups was a cough, with different frequencies that varied from 5 to 6 out of the given number of procedures altogether. Hypoxia and hypotension exercised a minor role across the groups, as just intermittent events were reported. Yao et al. (2021) reported a few cases out of 20, hypoxia, bradycardia, and hypotension in both the control group and the lidocaine group. Hayes et al. (2018) presented data for each dose group, reporting the duration of apnea for participants receiving Ketamine at doses of 0 mg/kg, 0.25 mg/kg, 0.5 mg/kg, and 1 mg/kg. The values were 59, 45, 57, and 39, respectively. Moreover, dizziness happened during the study and 6 out of 14 participants in the group at a concentration of 0 mg/kg, 7 out of 14 in the 0.25 mg/kg group, 8 out of 13 in the 0.5 mg/kg group, and 9 out of 14 in the 1 mg/kg group experienced these negative effects.  Karacaer et al. (2018) 34 participants reported 2 cases of nausea/vomiting, 1 case of hypoxia, and 2 cases of vomiting in the Remifentanil and ketamine, but no adverse effects have been reported in the Propofol and Ketamine groups. Bedirli et al. (2011) showed responses of the participants to fentanyl with propofol, of which 4 out of 40 experienced hypoxia, 7 out of 40 experienced bradycardia, and only 1 out of 40 experienced vomiting. Similarly, participants under propofol with tramadol did not report any cases of hypoxia. Moreover, one of the 40 participants experienced bradycardia, and three of them were with vomiting. Disma et al. (2005) for participants receiving propofol alone (Group P), 2 out of 80 participants experienced apnea, 1 out of 80 experienced tachycardia, 3 out of 80 experienced laryngeal spasm, and cough was observed in 6 out of 80 participants. In the group receiving propofol with midazolam (Group PM), 1 out of 78 participants experienced tachycardia, and 2 out of 78 experienced coughs. For participants receiving propofol with fentanyl (Group PF), 2 out of 82 participants experienced apnea. No other event was reported in this group. Paspatis et al. (2006) noted no complications in either the Oral Midazolam as a Premedication and Intravenous Propofol group or the Intravenous Propofol Alone group. Amer et al. (2020) reported in both the Dexmedetomidine-ketamine and Propofol-ketamine groups only nausea 4 out of 60 and 2 out of 60 respectively. Sienkiewicz et al. (2015) observed no complications in the Midazolam but in Propofol groups only 1 out of 26 experienced apnea. Rafeey et al. (2009) documented no adverse events in either the Oral or IV Midazolam groups. Ali et al. (2004) reported no adverse events in both the Fentanyl and Meperidine groups. |
| --- | --- |
| ESM. 3B. **Complications after the procedure** | Post-procedural effects after sedation were documented and appraised. In Tosun et al., (2007) with PK (Propofol/Ketamine) and PF (Propofol/fentanyl) groups, no adverse events were reported. Also, Ustun et al. didn’t notice any adverse event after the procedure either in Ketamine/propofol or Tramadol/propofol, But in Ulas (2016) study with a total of 119, cases of vomiting 2 in Midozolam/ketamine and 40 in Fentanyl/propofol were recorded, along with cases of visual disturbance (diplopia, nystagmus, etc.) 10 in Midozolam/ketamine and 87 in Fentanyl/propofol) and for headache adverse effects 23 in Midozolam/ketamine and 9 in Fentanyl/propofol lastly, 2 cases of hallucinations in Midozolam/ketamine and 18 cases in Fentanyl/propofol. As have other authors, Wang et al. conducted a study at different doses of S-ketamine, between 0 and 0.7 mg/kg, having 30 participants for each group. Adverse events were still reported among different dosage groups, although the frequencies of their occurrence were different. Group P (placebo: 0 mg/kg) comprised 1 case of vomiting, 2 cases of visual disturbances,8 cases of dizziness, and 3 cases of headache of 30 participants. There were 12 cases of dizziness, 1 case of headache, and 8 cases of visual disturbance in the S0.3 (S-ketamine 0.3 mg/kg) group out of a total of 29 sample sizes. Additionally, subjects of Group S0.5 (S-Ketamine 0.5 mg/kg) reported 13 cases of dizziness,1 case of headache, and 7 cases of visual disturbances. To conclude, Group S0.7 (S-Ketamine 0.7 mg/kg) managed to report 22 cases of dizziness as well as 5 cases of visual disturbance out of 30 individuals. Karacaer et al. mentioned nausea/vomiting in two cases of Remifentanil and ketamine, unlike in propofol and ketamine cases that revealed no adverse event. Sienkiewicz et al. & Bedirli et al. not mentioning any adverse effects of midazolam or propofol, and fentanyl or Tramadol combined with propofol. Rafeey et al. demonstrated that oral or intravenous midazolam was well tolerated without any adverse events, Ali et al. did not disclose any side effects from fentanyl or meperidine. Disma et al. had no adverse events with propofol alone, propofol with midazolam, and propofol with fentanyl. Lastly, Paspatis et al. did not report any adverse event with midazolam given as a premedication and propofol either intravenously or intravenous propofol alone. |

# ESM. 4. GRADE assessment of included outcomes


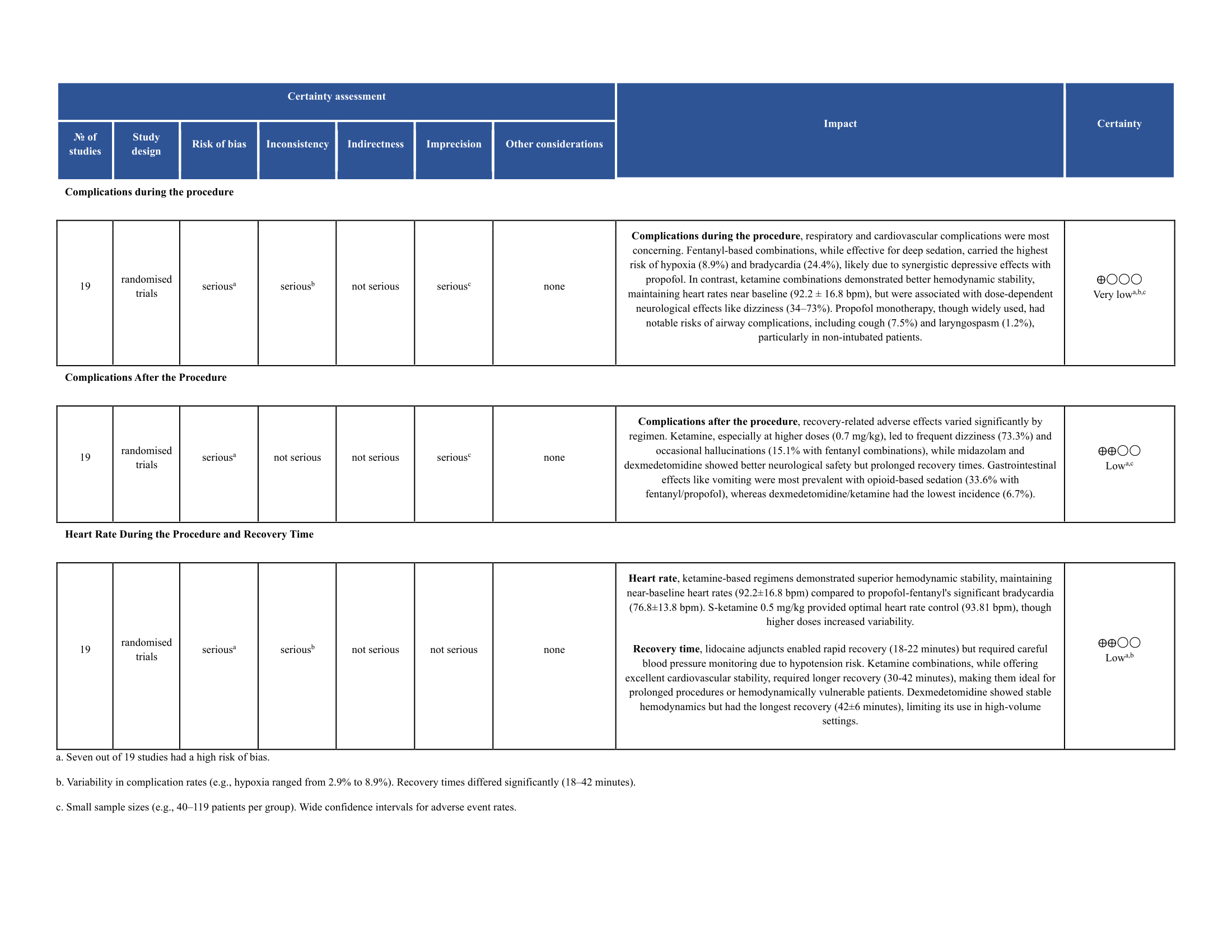

Supplement: Supplementary file 1 — (DOCX 547 KB) [file 210_2025_4557_MOESM1_ESM.docx]
